# Iron- and Hepcidin-Independent Downregulation of the Iron Exporter Ferroportin in Macrophages during *Salmonella* Infection

**DOI:** 10.3389/fimmu.2017.00498

**Published:** 2017-05-01

**Authors:** Alexandra Willemetz, Sean Beatty, Etienne Richer, Aude Rubio, Anne Auriac, Ruth J. Milkereit, Olivier Thibaudeau, Sophie Vaulont, Danielle Malo, François Canonne-Hergaux

**Affiliations:** ^1^Institut de Chimie des Substances Naturelles, Centre National de la Recherche Scientifique – UPR 2301, Gif-sur-Yvette, France; ^2^Department of Human Genetics, McGill University, Montréal, QC, Canada; ^3^McGill University Research Centre on Complex Traits, McGill University, Montréal, QC, Canada; ^4^IRSD, Université de Toulouse, INSERM, INRA, ENVT, UPS, Toulouse, France; ^5^Anatomie-Cytologie Pathologiques, CHU Bichat-Claude Bernard, Paris, France; ^6^INSERM, U1016, Institut Cochin, Paris, France

**Keywords:** *Salmonella* infection, anemia of inflammation, iron homeostasis, macrophage iron recycling, the iron regulatory hormone hepcidin, the iron exporter ferroportin

## Abstract

Retention of iron in tissue macrophages *via* upregulation of hepcidin (HAMP) and downregulation of the iron exporter ferroportin (FPN) is thought to participate in the establishment of anemia of inflammation after infection. However, an upregulation of FPN has been proposed to limit macrophages iron access to intracellular pathogens. Therefore, we studied the iron homeostasis and in particular the regulation of FPN after infection with *Salmonella enterica* serovar Typhimurium in mice presenting tissue macrophages with high iron (AcB61), basal iron (A/J and wild-type mice), or low iron (*Hamp* knock out, *Hamp*^−/−^) levels. The presence of iron in AcB61 macrophages due to extravascular hemolysis and strong erythrophagocytosis activity favored the proliferation of *Salmonella* in the spleen and liver with a concomitant decrease of FPN protein expression. Despite systemic iron overload, no or slight increase in *Salmonella* burden was observed in *Hamp*^−/−^ mice compared to controls. Importantly, FPN expression at both mRNA and protein levels was strongly decreased during *Salmonella* infection in *Hamp*^−/−^ mice. The repression of *Fpn* mRNA was also observed in *Salmonella*-infected cultured macrophages. In addition, the downregulation of FPN was associated with decreased iron stores in both the liver and spleen in infected mice. Our findings show that during *Salmonella* infection, FPN is repressed through an iron and hepcidin-independent mechanism. Such regulation likely provides the cellular iron indispensable for the growth of *Salmonella* inside the macrophages.

## Introduction

Human infectious diseases are still a major public health problem in particular because of the development of antibiotic resistance, the lack of new products, and the demise of antibacterial drug discovery by pharmaceutical companies (1). Such context leads to the emergence and reemergence of infectious diseases, and it becomes critical to develop alternative approaches to identify new antibacterial drugs and to propose new treatments. Therefore, the natural host defense mechanisms against invading microbes and the mechanisms regulating the virulence of microorganisms need to be better understood. An important host defense strategy against infection, known as “nutritional immunity,” relies on the sequestration of essential molecules, such as iron, preventing the growth of pathogens (2). Iron is essential for both the host and the invading microbes and plays a critical role in host–pathogen interactions. In response to infection, patients commonly develop hypoferremia (i.e., a decrease of iron in the circulation), a host response to limit iron availability to invading pathogens (3). However, for the host, hypoferremia also contributes to the establishment of the so-called anemia of inflammation (AI) (4), an anemia difficult to treat and that can add substantially to the morbidity of the underlying infection.

Iron sequestration in macrophages is a described hallmark of the AI and is an efficient mechanism to quickly deplete iron in the serum to limit the growth of extracellular pathogens (5). Two molecules, namely hepcidin (HAMP) and ferroportin (FPN), have been identified to play key roles in decreasing systemic iron level by promoting macrophage iron sequestration during infection (5). FPN is the only known mammalian iron exporter and is expressed at the cell surface of macrophages (6, 7). FPN is quickly downregulated through endocytosis and degradation upon interaction with HAMP (6, 8). HAMP is produced mainly by hepatocytes in case of inflammation and also by infected macrophages (9). Therefore, decreasing the expression of FPN to retain iron inside the macrophages could limit serum iron access to extracellular pathogens.

On the other hand, macrophages are a common niche for the replication of numerous intracellular pathogens including *Salmonella*. Increased iron level inside macrophages might therefore represent either an advantage for the growth of intracellular microorganisms or a host strategy to fight against intracellular bacteria through the generation of highly toxic reactive oxygen species *via* Fenton’s reaction (3). Recently, some studies have challenged these concepts and have suggested that macrophages infected with intracellular bacteria respond by decreasing their iron content *via* an upregulation of FPN to limit the growth of the invading microbes (10–12).

Therefore, the modulation of host iron homeostasis, in particular in macrophages, in response to infection with intracellular pathogens is currently a matter of debate, and the regulation of FPN is an important iron response to be evaluated in different intracellular bacterial infection settings. *Salmonella* is the most common bacterial cause of foodborne outbreaks, and many *Salmonella* strains are resistant to antibiotics. The main purpose of the current work was to explore the interplay between infection with *Salmonella* (*Salmonella enterica* serovar Typhimurium) and the systemic and macrophage iron homeostasis in different mouse models presenting distinct systemic and macrophages iron levels.

## Materials and Methods

### Animals and *Salmonella* Infection *In Vivo*

The generation of AcB61 was reported previously (13). A/J mice were purchased from the Jackson Laboratory. Hamp^tm1Svl^ knockout mice [*Hamp*^−/−^ (14)] were transferred onto a 129S6 background (129S6.B6*129S2-*Hamp^tm1Svl^*). Both female and male aged between 8 and 12 weeks were used for the current study. The mice were fed with the diet Teklad 2920X, which contains 200 mg/kg of iron. All the experiments were done under the same housing conditions at McGill University (Montreal, QC, Canada). *In vivo* intravenous infections [~1,000 CFUs for A/J and AcB61 and ~5,000 CFUs for wild-type (WT) and *Hamp*^−/−^] were performed with *S. enterica* serovar Typhimurium (strain Keller) as previously described (15).

### Macrophage Cultures and *Salmonella* Infection *In Vitro*

Murine bone marrow-derived macrophages (BMDMs) from CD1 mice were cultured as previously described (16). *In vitro* infection of macrophages (MOI of 5–10) was performed for 1 h with *S. enterica* serovar Typhimurium (strain SL1344) (17). Extracellular bacteria were killed by incubation with 100 µg/ml gentamicin in fresh medium for 1 h. Cells were then washed and cultured in fresh medium containing 10 µg/ml gentamicin until the time points of RNA extraction.

### Blood Parameters Analysis

Hematology profiles were performed at the McGill Comparative Medicine and Animal Resources Centre (Montréal, QC, Canada). Plasma iron, ferritin, transferrin, and bilirubin levels were measured with an Olympus AU400 automat at the Laboratory of Biochemistry at the Institut Fédératif de Recherche 02, CHU Bichat-Claude Bernard (Paris, France).

### Tissues Iron Studies

Liver and spleen iron contents were determined by acid digestion (18) and measured with an Olympus AU400 automat. Tissue iron staining was done using Perls’ Prussian blue solution and examined under a light microscope and photographed or digitized using a slide scanner (Pannoramic 250 from 3DHISTEC).

### Immunohistofluorescence Studies

After blocking (1% BSA and 10% heat inactivated goat serum) for 30 min at room temperature, deparaffinized tissues sections were incubated with primary antibodies for 1 h: rabbit anti-FPN (7, 19): 1/50 to 1/100; rabbit anti-HMOX1 (Stressgen): 1/500; rat anti-F4/80 (AbDserotec): 1/500. After three washes with PBS/0.5% BSA, sections were incubated for 1 h at RT with Goat anti-rabbit-alexa488 (1/200) and Goat anti-rat-Alexa563 (1/200) (MolecularProbes). After mounting, sections were visualized using either an epifluorescence microscope LEICA DM-IRM or a Zeiss confocal fluorescent microscope. Images were acquired using either ARCHIMED-PRO (Microvision Instruments) or Zeiss LSM Image Browser softwares.

### Western Blot Analysis

Crude membrane fractions (40 µg for spleen and 80 µg for liver) from mouse tissues were prepared and analyzed by western blotting as previously described (7). Antibodies were diluted in blocking solution as follows: anti-FPN (7, 19): 1/200 (liver) or 1/500 (spleen), anti-HMOX1 (Stressgen): 1/4,000, anti-LAMP1 (DSHB): 1/500, and anti-TfR1 (Zymed): 1/200.

### RNA Studies

Complementary DNAs were synthesized from total RNA (Trizol) isolated from tissues or BMDM and using M-MLV reverse transcriptase (Invitrogen). Quantitative PCR was performed on Chromo4 Real-Time PCR Detection System (Bio-Rad Laboratories) or LightCycler 480 Instrument (Roche Diagnostics) using Brilliant SYBR Green QPCR Master Mix (Stratagene). Gene expression fold changes were calculated using the formula 2^−ΔΔCt^, in which ΔΔCt^A–B^ = (Ct^gene^ − Ct^Hprt^) B − (Ct^gene^ − Ct^Hprt^) A and A = WT and B = *Hamp*^−/−^. For tissues, data are presented as fold changes (2^−ΔΔCt^) in infected mice relative to the mean value of A/J or WT (control) at each time point. For BMDM, the gene *Hprt* was used as a reference gene, and relative gene expression is expressed in −ΔCT (CT gene of interest − CT *Hprt*).

### Statistical Analysis

Except for CFUs (unpaired two-tailed Student’s *t*-test), data were analyzed by two-way ANOVA using Sidak’s multiple comparisons test followed by unpaired *t*-tests. GraphPad Prism version 6 was used for statistical analysis (GraphPad Software, La Jolla, CA, USA).

## Results

### Impact of *Salmonella* Infection on Anemia and Iron Homeostasis in A/J and AcB61 Mice

**Figure 1:**
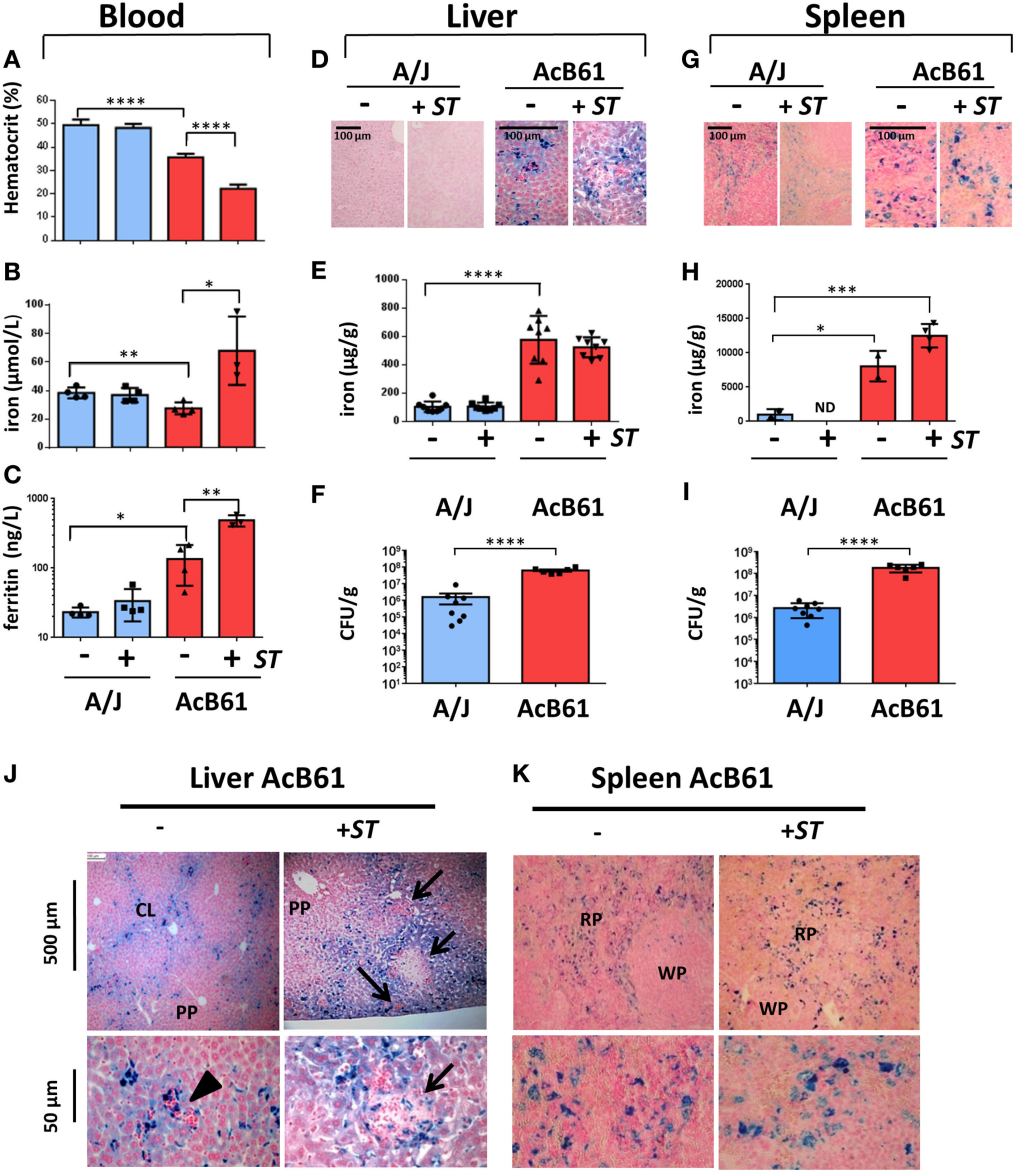
**Infectious, hematological and iron metabolism parameters in A/J and AcB61 mice following infection with *Salmonella* Typhimurium (*ST*)**. Blood parameters from A/J and AcB61 including hematocrit **(A)**, iron **(B)**, and ferritin **(C)** were analyzed before (−) or after 5 days of infection with *ST* (+*ST*). Iron levels were assessed by Perls staining **(D,G)** and dosage **(E,H)** on liver **(D,E)** or splenic **(G,H)** tissues. Bacterial load at day 5 of infection with *ST* was studied by measuring CFUs in liver **(F)** and spleen **(I)** of infected A/J and AcB61 mice. Statistical significance: *P < 0.05, **P < 0.01, ***P < 0.001, ****P < 0.0001. ND, not determined. Hepatic **(J)** and splenic **(K)** iron distribution in AcB61 mice before and after *Salmonella* infection. Liver and spleen sections from naive AcB61 mice (−) or AcB61 mice infected with *ST* (+*ST*, Day 5) were processed for Perls staining. Arrowhead indicates the presence of erythropoeitic islands in naïve AcB61 liver attesting of extramedullar erythropoiesis in these mice. After infections, numerous inflammatory foci and pathological lesions were observed in iron-rich region of the liver (arrows). Strong iron accumulation was seen in splenic macrophages of the RP before and after *Salmonella* infection. PP, periportal vessels; CL, centrilobular vessels; RP, red pulp; WP, white pulp. *n* = 4–8 mice per genotype used for the different measurements. The data are presented as mean ± SD.

The recombinant congenic mouse strain AcB61 was generated from A/J and C57BL/6J mice and presents a deficiency in red blood cell pyruvate kinase activity (*de novo* mutation in *Pklr*). As a consequence of this mutation, AcB61 mice present chronic hemolytic anemia with tissue iron overload (20–22). AcB61 and their parental controls (A/J mice) were infected intravenously with *Salmonella* Typhimurium (*ST*), and samples were collected before (D0) and 5 day postinfection (D5). Hematocrit (A), plasma iron (B), and ferritin levels (C) in blood were analyzed (Figure 1). Consistent with previous reports (22), AcB61 mice showed a constitutive anemia at D0 with a lower hematocrit (35% in AcB61 versus 50% for A/J) that worsens during *Salmonella* infection (~20% in AcB61; Figure 1A). Signs of anemia occurred later during infection in A/J mice (data not shown). Compared to A/J, AcB61 mice presented hypoferremia (Figure 1B) and hyperferritinemia (Figure 1C). With *Salmonella* infection, both plasma iron and ferritin levels increase significantly in AcB61.

In the liver at D0, Perls staining of tissue sections (Figure 1D) and quantitative determination of iron (Figure 1E) indicated a strong iron accumulation in AcB61 liver when compared to A/J liver. We did not observe any significant changes in iron levels in the liver of both A/J and AcB61 after infection. Importantly, the bacterial load was significantly higher in the liver of AcB61 mice when compared to A/J (Figure 1F). In the AcB61 spleen, the iron level was significantly higher than the one detected in A/J and tended to slightly increase with infection (Figure 1H) with a marked iron accumulation in enlarged splenic macrophages (Figure 1G). On the other hand, the Perls staining of A/J spleen suggested a slight decrease in iron after infection (Figure 1G). As observed in the liver, the bacterial load was significantly higher in the spleen of AcB61 mice when compared to A/J (Figure 1I).

We next analyzed more precisely the localization of iron in both liver (Figure 1J) and spleen (Figure 1K) from AcB61 before and after infection. Histological examination of Perls staining indicated iron accumulation mostly in sinusoid zones and centrilobular (CL) area of the naïve AcB61 liver, whereas most periportal (PP) zones were not stained (Figure 1J). At the cellular level, iron strongly accumulated (deep blue) in Kupffer cells with some milder iron staining (light blue) in surrounding hepatocytes (Figure 1J, lower panels; Figure S1A in Supplementary Material). Signs of extramedullary erythropoiesis (clusters of nucleated cells surrounding or near iron loaded macrophages) were also observed at the vicinity of vessels in uninfected AcB61 (Figure 1J, arrowhead). With *Salmonella* infection, numerous and enlarged inflammatory foci were observed in iron-rich regions of the AcB61 liver (Figure 1J, arrows).

Histologic examination of the spleen of AcB61 mice before infection revealed a strong expansion of the red pulp (RP) and evidence of extramedullary erythropoiesis with numerous trapped RBC (Figure 1K, lower panel). Important accumulation of iron in the AcB61 spleen was clearly detected in enlarged splenic macrophage of the RP before and after infection (Figure 1K; Figure S1A in Supplementary Material).

Several observations in AcB61 mice including the presence of ingested RBC, the strong expression of the heme oxygenase 1 (heme catabolism enzyme), splenomegaly, and an high bilirubin level (marker of erythrophagocytosis and heme iron recycling) indicate that the macrophage iron overload of AcB61 mice is due to extravascular hemolysis and a strong erythrophagocytosis (EP) activity in tissue macrophages (Kupffer and splenic) (Figure S1 in Supplementary Material). FPN protein was also strongly expressed in AcB61 tissues (Figure S1E in Supplementary Material) and was localized at the cell surface of both hepatic (Figure S1F in Supplementary Material) and splenic (Figure S1G in Supplementary Material) AcB61 macrophages, presenting numerous engulfed RBC. Altogether our observation indicates a strong clearance of red blood cells and heme catabolism by macrophages in AcB61. As a consequence, such erythrophagocytosing AcB61 macrophages present large amount of iron and a strong expression of FPN.

### FPN Expression in A/J and AcB61 after *Salmonella* Infection

**Figure 2:**
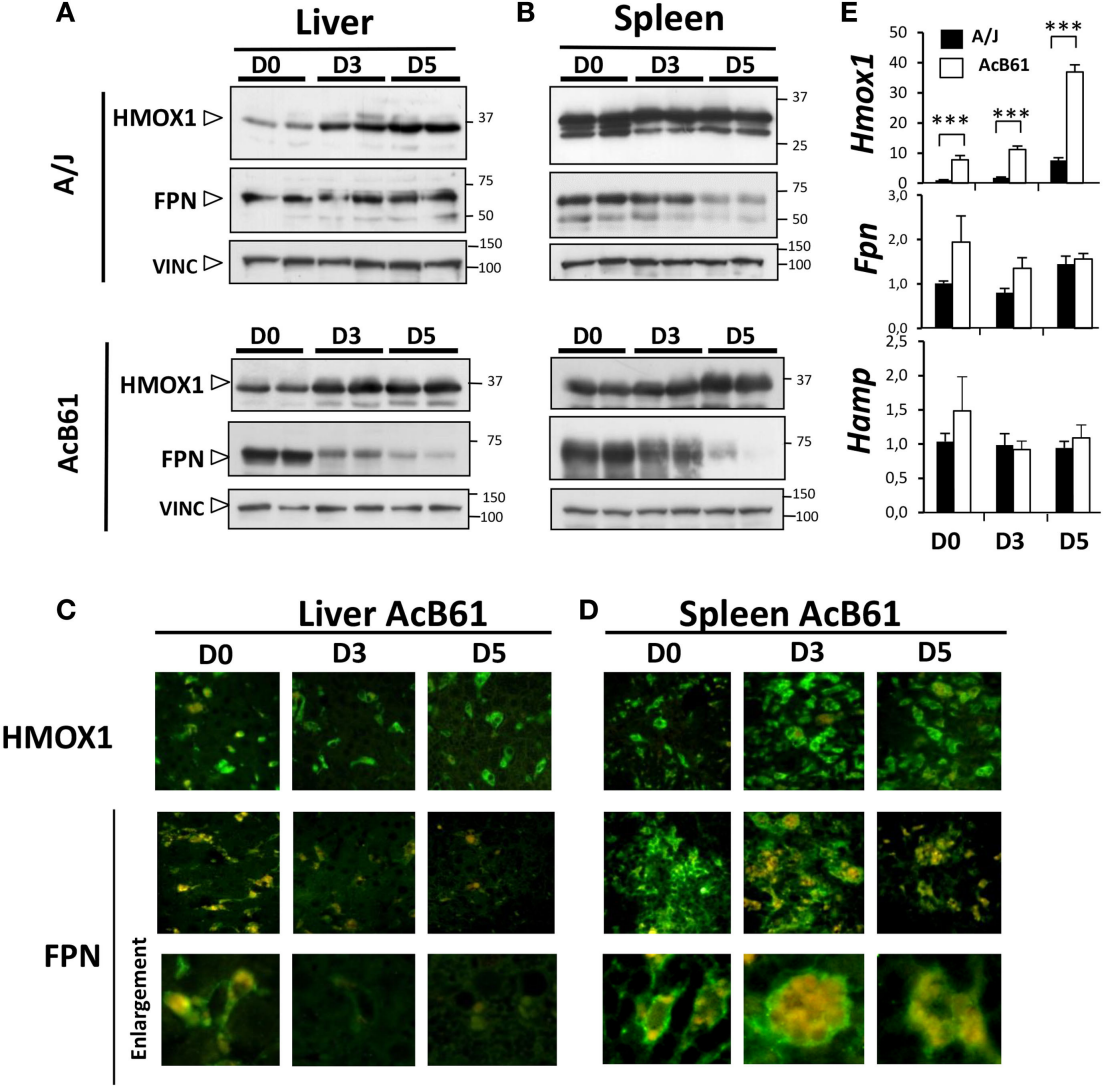
**Ferroportin (FPN) and HMOX1 expression during *Salmonella* infection in A/J and AcB61 mice**. **(A,B)** Western blot analysis of HMOX1, FPN, and VINC (loading control) expression in liver **(A)** and spleen **(B)** during *Salmonella* Typhimurium (*ST*) infection in both A/J and AcB61. **(C,D)** Immunofluorescence labeling (green) of FPN or HMOX1 and autofluorescence (red) of RBC hemoglobin in liver of *Salmonella*-infected AcB61 mice. During *Salmonella* infection, HMOX1 expression was maintained, whereas FPN expression at the cell surface of tissue macrophages cells strongly decreased. **(E)** RT-qPCR analysis of *Fpn, Hmox1*, and *Hamp* mRNA expression in A/J (*n* = 4) and AcB61 (*n* = 4) liver before and after *ST* infection. Data are representative of three independent experiments. D0, uninfected; D3 and D5, 3 and 5 days postinfection, respectively. Statistical significance: ***P < 0.001. The data are presented as mean ± SD.

During *Salmonella* infection, protein expression of HMOX1 increased in the liver (Figures 2A,C) of both of A/J and AcB61 mice and in the spleen (Figures 2B,D) of AcB61 mice. HMOX1 expression was maintained in spleen of infected A/J (Figure 2B). On the other hand, infection leads to a profound downregulation of FPN in the liver (Figure 2A, lower panels) and in the spleen (Figure 2B, lower panels) of AcB61. FPN downregulation was also observed in the spleen of A/J mice (Figure 2B, upper panels). Decreased expression of FPN in AcB61 organs was associated with the disappearance of FPN at the cell surface of tissue macrophages (Figures 2C,D). During infection, we also observed strong EP activity in the spleen of AcB61 as illustrated by the high number of RBC (Hb autofluorescence) in large splenic macrophages (Figure 2D, enlargement).

At the hepatic mRNA levels (Figure 2E), in uninfected mice, *Fpn* and *Hmox1* were increased in AcB61 liver when compared to A/J tissues. *Hmox1* mRNA expression progressively increased during infection in both A/J and AcB61 liver. Contrasting with the strong protein downregulation, no major changes of *Fpn* mRNA expression were observed in AcB61 liver during *Salmonella* infection. In addition, *Hamp* expression did not change significantly during infection, suggesting that HAMP may not contribute to the anemia and downregulation of FPN during *ST* infection in AcB61 mice.

### Impact of *Salmonella* Infection on Anemia and Iron Homeostasis in WT and *Hamp*^−/−^ Mice

To understand better the role of HAMP during *Salmonella* infection, we next studied the impact of *Salmonella* infection in mice deficient in HAMP (*Hamp*^−/−^) (14). *Hamp*^−/−^ mice have been shown to develop a specific iron phenotype with high serum iron concentration, excess iron deposition in hepatocytes, and low iron levels in tissue macrophages (14).

**Figure 3:**
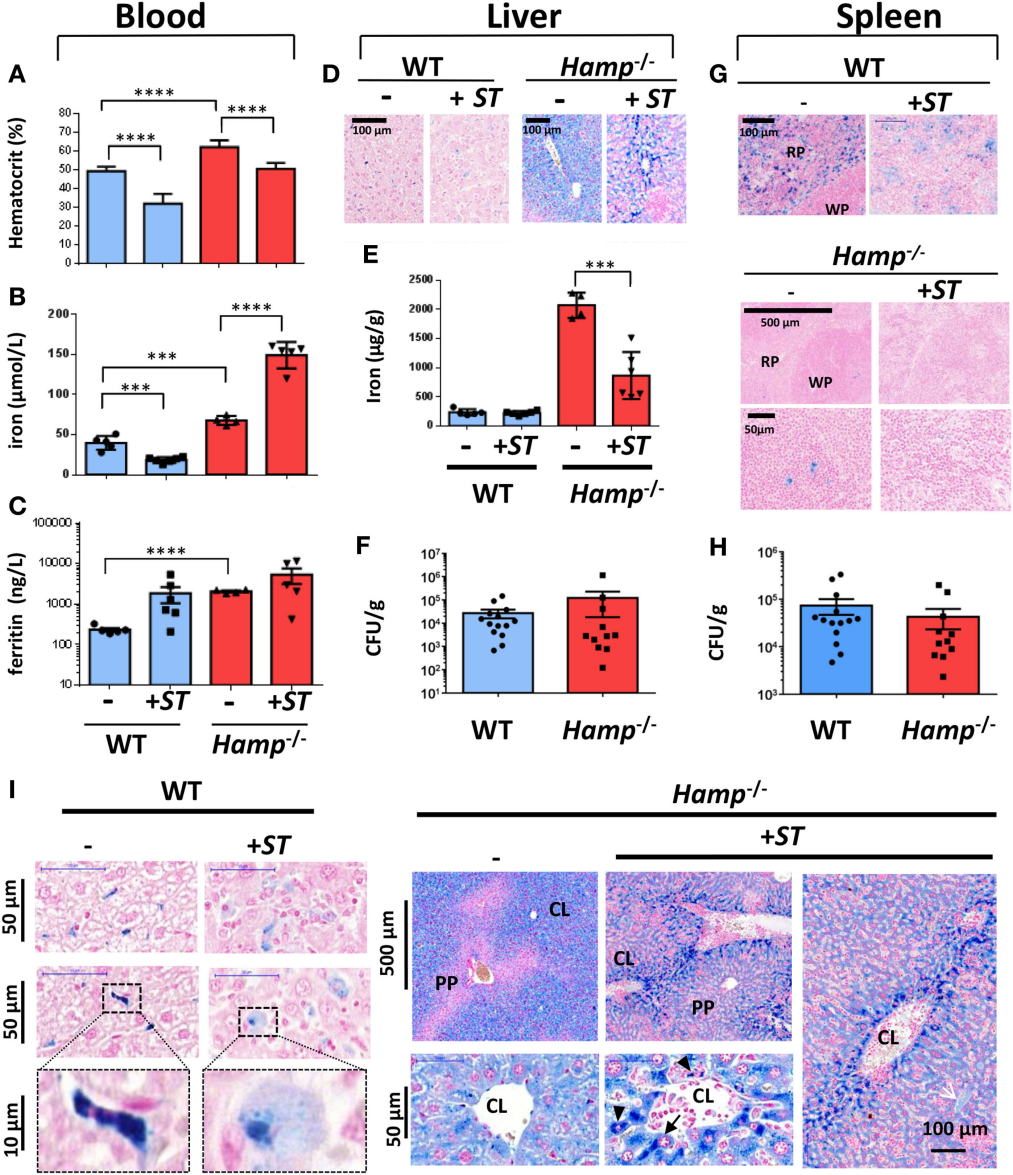
**Infectious, hematological and iron metabolism parameters in wild-type (WT) and hepcidin knockout (*Hamp*^−/−^) mice following infection with *Salmonella* Typhimurium (*ST*)**. Blood parameters from WT and *hepcidin* knockout *Hamp*^−/−^ including hematocrit **(A)**, iron **(B)**, and ferritin **(C)** were analyzed before (−) or after 10 days of infection with *ST* (+*ST*). Iron levels were assessed by Perls staining **(D,G)** and dosage **(E)** on liver **(D,E)** or splenic **(G)** tissues. Bacterial load at day 10 of infection with *ST* was studied by CFUs in the liver **(F)** and spleen **(H)** of infected WT and *Hamp*^−/−^ mice. Statistical significance: ***P < 0.001; ****P < 0.0001. **(I)** Hepatic iron localization in WT and *Hamp*^−/−^ mice before and after *Salmonella* infection. Perls staining of liver sections from naive (−) mice or mice infected with *ST* (+*ST*, Day 10). Lower panels in WT mice show high magnification of the cellular localization of iron in Kupffer cells. PP, periportal vessels; CL, centrilobular vessels. *n* = 4–15 mice per genotype. The data are presented as mean ± SD.

Wild-type and *Hamp*^−/−^ mice were intravenously infected with *ST* (+*ST*; Figure 3). After 10 days postinfection, WT mice present signs of anemia with hematocrit levels below 40% (Figure 3A) and decreased plasma iron levels (Figure 3B). As previously described (14), *Hamp*^−/−^ mice present a higher hematocrit (60%) and a higher concentration of iron and ferritin in the blood when compared to WT (Figures 3A–C). During *Salmonella* infection in *Hamp*^−/−^ mice, the hematocrit significantly decreased to 50% but was accompanied with a significant increase (around two times more) of plasma iron (Figures 3A,B).

With infection, no major changes in liver iron level were observed in WT mice (Figures 3D,E). On the other hand, iron was strongly detected in *Hamp*^−/−^ liver (around 2,000 µg/g) and decreased significantly by more than 50% after infection (Figure 3E). In parallel to the hepatic iron overload phenotype of *Hamp*^−/−^ mice, we did not observe significant changes in bacterial CFUs in the liver (Figure 3F) of *Hamp*^−/−^ versus WT mice after *Salmonella* infection.

In the *Hamp*^−/−^ spleen, iron was strongly depleted in the macrophages of the RP when compared with WT (Figure 3G) corroborating the described low iron level of *Hamp*^−/−^ macrophages. In WT spleen, Perls staining indicated a decrease in macrophage iron after *Salmonella* infection (Figure 3G; Figure S2 in Supplementary Material). As observed in the liver, we did not detect significant changes in bacterial CFUs (Figure 3H) in *Hamp*^−/−^ versus WT spleen. Together, these data suggest that the serum and parenchymal iron overload phenotype of *Hamp*^−/−^ mice does not favor the growth of *Salmonella in vivo*.

### Changes in Iron Localization in *Hamp*^−/−^ Liver during *Salmonella* Infection

A careful microscopy analysis of Perls staining confirmed the presence of iron (deep blue staining) in WT Kupffer cells, which tend to decrease (light blue staining) after *Salmonella* infection (Figure 3I). In the liver of uninfected *Hamp*^−/−^ mice, iron accumulation was observed in hepatocytes of CL zones, whereas PP areas were not stained (Figure 3I). Interestingly, a change in the cellular localization of iron was observed after *Salmonella* infection with higher iron concentration in macrophages (Figure 3I, arrowheads) and hepatocytes (Figure 3I, arrows) lining the CL zones and the sinusoids walls. The decrease in liver iron content and its redistribution during infection suggest that *Salmonella* alter mechanisms of iron storage or export.

### Downregulation of FPN during Infection by *Salmonella* Is Independent of Hepcidin

**Figure 4:**
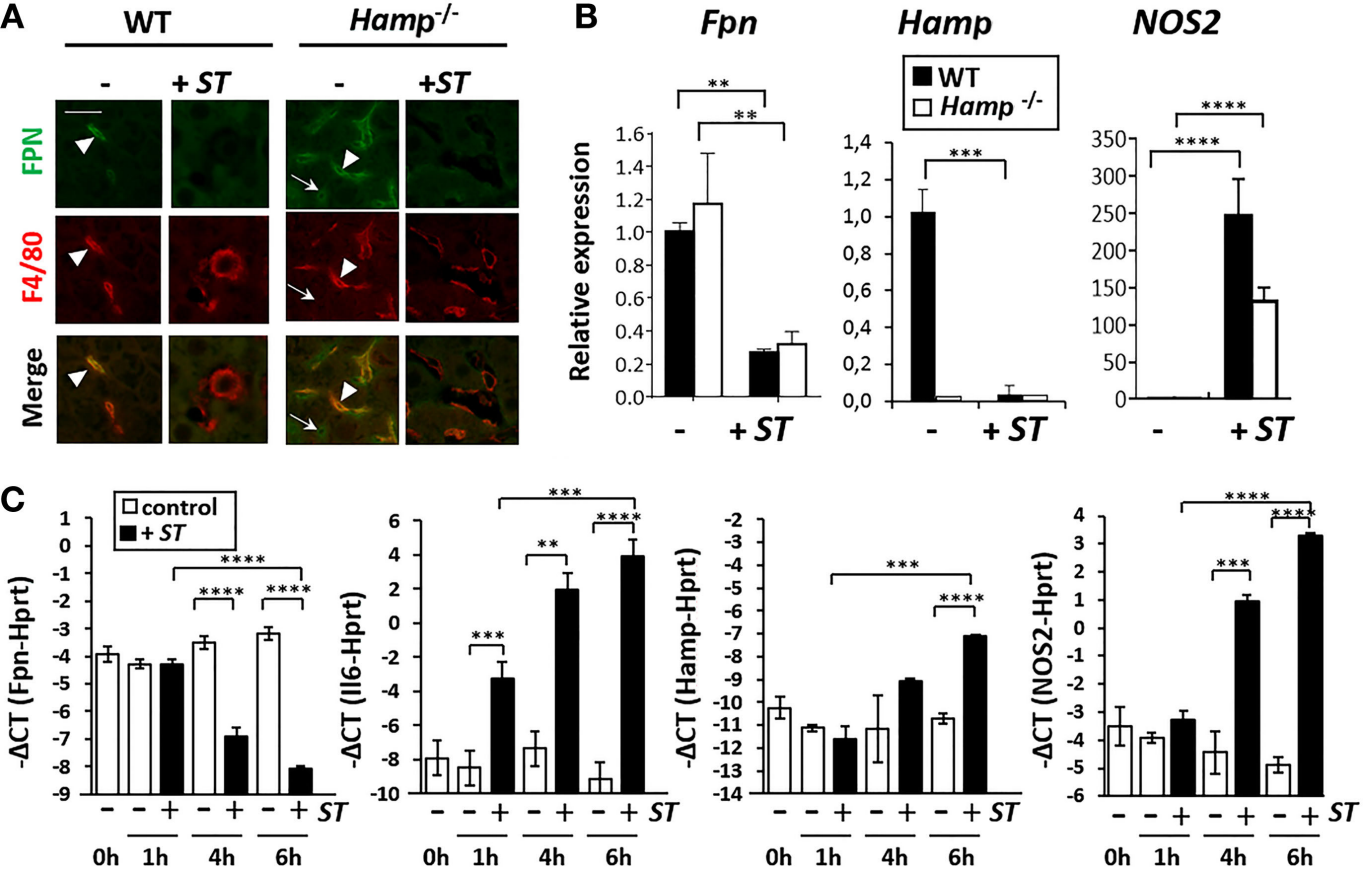
**Hepcidin-independent downregulation of ferroportin (FPN) expression in liver and macrophages after *Salmonella* infection**. **(A)** Immunohistofluorescence of FPN (green) and F4/80 (macrophage marker; red) expression in liver of naïve mice (−) or mice infected with *Salmonella* Typhimurium (*ST*) (+*ST*). **(B)** RT-qPCR analysis of *Fpn, Hamp*, and *nitric oxide synthase 2* (*NOS2*) mRNA expression in wild-type (WT) (black bars) and *Hamp*^−/−^ (white bars) liver before (−) and after (+*ST*) *ST* infection. Data are presented as fold changes (2^−ΔΔCt^) in infected mice relative to the mean value of WT (control) at each time point. **(C)** RT-qPCR analysis of *Fpn, IL6, Hamp*, and *NOS2* mRNA expression in bone marrow-derived macrophage (BMDM) at different time points after infection with *ST*. The relative gene expression is expressed as −ΔCt (Ct^gene of interest^ − Ct^Hprt^). White bars: uninfected BMDM (−); black bars: infected BMDM (+). Statistical significance: **P < 0.01; ***P < 0.001; ****P < 0.0001. The data are presented as mean ± SD.

To determine whether FPN is involved in the redistribution of iron in the absence of *Hamp*, we measured FPN expression during *Salmonella* infection in *Hamp*^−/−^ mice. As previously described, *Hamp*^−/−^ mice expressed high levels of FPN protein in both spleen (Figure S3 in Supplementary Material) and liver (Figure 4A) compared to WT mice. FPN was mostly detected in Kupffer cells (F4/80+) in WT liver, whereas in *Hamp*^−/−^ liver, FPN was strongly expressed by both Kupffer cells (F4/80^+^) and hepatocytes (F4/80^−^) (Figure 4A). After *ST* infection, in both WT and *Hamp*^−/−^, the expression of FPN was strongly decreased in hepatocytes and Kupffer cells when compared to uninfected tissues (Figure 4A). Similarly, a decrease of FPN expression in macrophages of the RP in WT and *Hamp*^−/−^ spleen was observed after *ST* infection (Figure S3 in Supplementary Material). Similar observation was made after *Salmonella* Enteritidis (*SE*) infection (Figure S4 in Supplementary Material). In some microscopy fields of the liver, despite a global decrease of FPN staining in most of the section area, some localized FPN- and F4/80-positive regions were detected after *ST* (Figure S5 in Supplementary Material) and *SE* (Figure S4 in Supplementary Material) infections. Panel B in Figure S5 in Supplementary Material clearly indicated, within infected liver, the presence of large resident Kupffer cells negative for FPN expression (F4/80^+^; arrowhead) with smaller and round recruited monocytes both positive for FPN and F4/80 (arrow dot).

In parallel to the decrease of FPN protein expression, the level of *Fpn* mRNA was significantly downregulated in both WT and *Hamp*^−/−^ liver after *ST* infection (Figure 4B). Previous reports (16, 23) suggest that the nitric oxide synthase 2 (NOS2) play a role in a positive regulation of FPN during *Salmonella* infection. However, concomitant with the decrease of *Fpn*, an increase in the mRNA expression of *Nos2* gene was observed with *ST* infection in both WT and *Hamp*^−/−^ liver (Figure 4B).

A time-dependent downregulation of *Fpn* mRNA was also observed in BMDM cultures infected with *ST* (Figure 4C). Such negative regulation of *Fpn* was rapid occurring after 4 h of infection with no significant changes of *Hamp* level at that time. In contrast to *in vivo* infections, *Hamp* expression was slightly but significantly upregulated at 6 h in BMDM during *Salmonella* infection, suggesting that downregulation of *Hamp in vivo* most likely reflects a global repression in hepatocytes. As a control of BMDM infection, *Il6* expression strongly increased during *ST* infection (Figure 4C). As observed in liver, *Nos2* was also induced in BMDM after *Salmonella* infection. Overall these data are consistent with the conclusion that decreased expression of FPN during *Salmonella* infection is independent of HAMP.

## Discussion

In this article, we characterized the interplay between iron homeostasis and intracellular *Salmonella* infection, using different mouse models presenting distinct systemic and macrophages iron contents. Indeed two distinct models were used presenting either macrophage iron overload (AcB61) or macrophage iron deficiency but systemic iron overload (*Hamp*^−/−^).

Among our models, the AcB61 mice were the most susceptible to *ST* infection. AcB61 mice harbor a mutation in the gene *Pklr* leading to PK deficiency and resulting in chronic hemolytic anemia and tissue iron overload (20–22). We observed intensive EP activity in AcB61 tissue macrophages *in vivo*, which is consistent with *in vitro* studies showing that *Pklr*-deficient erythrocytes were more vulnerable to phagocytosis by macrophages than control erythrocytes (24). As a consequence of enhanced EP activity in AcB61 macrophages, strong heme recycling is observed with increased bilirubinemia and enhanced expression of both HMOX1 and FPN. Heme is known to be a potent inducer of HMOX1 transcription (25), and both heme and iron positively regulate macrophage FPN at both transcriptional and posttranscriptional levels (23, 26). In addition, FPN was strongly detected *in vivo* at the cell surface of AcB61 hepatic and splenic macrophages, suggesting some export of the iron from the cytosol to circulation.

The important iron storage and iron fluxes in AcB61 macrophages likely represent an advantage for the growth of *Salmonella* and contribute to the high susceptibility of AcB61. Interestingly, AcB61 mice have been challenged for their response to infection with several intracellular bacteria including *Listeria monocytogenes* (D. Malo, unpublished), different strains of *Mycobacterium bovis* (22, 27), and *Legionella pneumophila* (13). For all these models of infection, the Pklr mutation in AcB61 did not contribute to the clinical phenotype, and no further studies focusing on iron metabolism were performed. Other observations (not shown) indicate that the exacerbated susceptibility of AcB61 mice to *Salmonella* infection is not the consequence of a blunted immune response or a defect in the expression of the iron-siderophore binding protein lipocalin 2 and therefore likely reflects the accessibility to intracellular iron by the bacteria. Iron is an indispensable metal for the spread of virtually all human pathogens (3). Of note, numerous *Salmonella*-induced granulomas, which represent infected foci containing the bacteria, were mostly localized in iron-rich regions in AcB61 mice. Importantly, there was a significant decrease of serum iron in AcB61 when compared to A/J, suggesting that despite the fact that *Salmonella* is a facultative intracellular bacterium, it takes more advantage of the intracellular macrophage iron sources rather than of the extracellular sources. Accordingly, in spite of a high iron level in blood and hepatocytes, we observed no change in *Salmonella* load in *Hamp*^−/−^ mice when compared to WT mice. However, we previously showed that mice deficient in Hamp were significantly more susceptible to lethal infection than heterozygous or wild type littermates (28). Such observation indicates the involvement of Hamp during systemic model of *ST* infection. However, the exact mechanism underlying such differences remains obscure.

Iron deficiency, bone marrow suppression, and hemolysis are described to participate in the establishment of anemia in infectious diseases (4). Lower hematocrit levels after *Salmonella* infection were observed in AcB61 and *Hamp*^−/−^ mice despite important levels of plasma iron, suggesting that these mice do not develop iron-restrictive anemia during infection. Our observations are concordant with literature, suggesting that the main driving force for the decrease of hematocrit during *Salmonella* infection is an accelerated clearance of RBC (29, 30). Indeed, during *Salmonella* infection, AcB61 spleens present signs of strong EP activity with numerous RBC per macrophages. *Salmonella* infection *via* the stimulation of Toll-like receptor 4 has been shown to stimulate macrophages to hemophagocytosis, a process that lead to the phagocytosis of red and white blood cells (29, 30).

In addition, our work suggests that the *Salmonella*-induced anemia is, at least in part, independent of HAMP. Indeed, either no changes (AcB61 mice) or a decrease (WT mice) of hepatic *Hamp* expression is associated with anemia after *ST* infection, and even in *Hamp*^−/−^ mice, a decreased hematocrit is still observed. *In vivo*, the expression of *Hamp* in the hepatocyte is governed by stimulatory (iron overload and inflammation) and inhibitory factors (erythropoietic ERFE and hypoxia), the net effect of these factors defining the *Hamp* level in the organism (31). In uninfected AcB61 mice, the positive iron regulator is likely compensated by the negative erythropoietic regulator (ERFE) leading to normal but inadequately low level of *Hamp* for the degree of iron loading observed in these mice. Indeed, we observed extramedullary erythropoiesis and increase of *Erfe* mRNA levels (not shown) in the spleen of AcB61. As previously observed (28), in WT mice, *Hamp* expression was repressed after *ST* infection. In contrast, other studies have reported increased expression of *Hamp* during ST infection in mice (32) or *Salmonella* Typhi in humans (33). Interestingly, in a model of the AI using a heat-killed Gram-negative bacteria, *Brucella abortus, Hamp* was shown to be upregulated in an early phase associated with erythropoietic suppression but was downregulated in a second later phase in parallel with an increase in EPO and erythropoiesis (34, 35). In addition, *Salmonella* infection has been shown to initiate extramedullary erythropoiesis and splenomegaly with increases in RBC precursors and EPO production (36). These differences observed between studies in the regulation of *Hamp* during *Salmonella* infection may rely on differences in the degree of compensatory erythropoiesis at the infection time (negative regulation of *Hamp via* ERFE).

In this study, we observed a strong negative regulation of the iron exporter FPN at both the mRNA and the protein level in *Hamp*^−/−^ mice, indicating that the repression of the iron exporter by *Salmonella* infection is independent of HAMP action. Recently, a strong HAMP-independent, negative regulation of FPN mRNA and protein was also documented in BMDM and liver and spleen of mice in response to acute inflammatory conditions induced by TLR2/6 agonists (37). In this study, the reduced expression of FPN in macrophages was sufficient to rapidly induce hypoferremia in mice (37). Similarly, reduction of spleen *Fpn* mRNA level by TLR4 agonist was shown to be HAMP independent (38).

In WT mice, we also observed a concomitant decrease in both FPN expression and plasma iron with a decrease in *Hamp* expression. Together, such observations suggest that beside HAMP effect, other mechanisms exist to induce a pathogen-mediated hypoferremic response, contributing to the AI.

*In vivo*, the negative regulation of FPN protein was observed in tissue macrophages after *ST* infection. Similar observation was made after *SE* infection (Figure S4 in Supplementary Material). Of note, we did not observe a decrease in *Fpn* mRNA expression in infected AcB61 mice despite the strong loss of FPN protein expression at the cell surface of AcB61 macrophages. The strong positive regulation of *Fpn* mRNA expression by heme and iron in erythrophagocytic AcB61 macrophages likely counteracts the *Salmonella*-mediated negative regulation at the level of mRNA. Such observation suggests that posttranscriptional regulations may exist since FPN protein expression is diminished without any changes of *Fpn* and *Hamp* mRNA levels. Recently, iron regulatory proteins (IRPs) have been shown to play a role during *Salmonella* infection (39). FPN contains an iron-responsive element in its 5′ UTR, and its translation is repressed by the IRPs. Therefore, during *Salmonella* infection, IRPs could block the translation of *Fpn* and thereby contribute to the decrease of FPN protein levels despite no alteration at the levels of mRNA. However in the context of AcB61 mice, the high iron content observed in macrophages likely impairs the action of the IRPs. Since FPN protein expression decreased despite maintained level of *Fpn* mRNA in AcB61 mice during salmonella infection, other posttranscriptional mechanism(s) may occurred.

Interestingly, FPN- and F4/80-positive cluster of cells were detected only in the liver of *Hamp* KO mice after *Salmonella* infection. In these mice, our cellular analysis strongly suggests that such cellular aggregates correspond to the recruitment of uninfected circulating monocytes overexpressing FPN because of the lack of Hamp.

*In vitro*, the negative FPN regulation was directly observed at the level of mRNA in *ST*-infected cultured BMDM. Our data are consistent with previous observations showing decreased FPN mRNA expression *in vivo* (37, 40) and *ex vivo* in cultured murine and human macrophages treated with lipopolysaccharide (LPS) (16, 41–43). *ST* infection and LPS stimulation were shown to induce similar changes in macrophage gene expression (44). The molecular mechanism of *Fpn* mRNA repression in macrophages *via* LPS/TLR4 stimulation is still not known. Moreover, downregulation of *Fpn* expression in macrophages was also reported with TLR2/6 (37, 45) expanding the FPN response to various pathogen-associated molecules.

In the context of intracellular pathogen infection, low levels of FPN in macrophages will favor cellular iron sequestration and bacterial growth inside the infected cells. This is consistent with *in vitro* studies showing that degradation of FPN resulted in increased macrophage bacterial growth in *Salmonella*-infected J774 macrophages (46). In opposition to this cellular scenario, other authors proposed that during infection with macrophage-tropic intracellular pathogens, macrophages respond by an upregulation of FPN to limit intracellular iron content (10, 12). Increases in FPN mRNA and protein expression in mouse macrophages cell lines RAW264.7 or thioglycollate-elicited peritoneal macrophages have been reported during *Salmonella* infection (10). The same authors have proposed that upregulation of FPN during *Salmonella* infection involves NO production by NOS2 (10, 12). However, in our *ST*-infected BMDM as well as in the liver of infected mice, the FPN gene repression occurred with a concomitant increase of the *NOS2* expression. The role of NOS2 and NO in the regulation of FPN needs further investigation. The discrepancy between studies regarding the regulation of FPN during intracellular infection is unclear and warrants continued effort to clarify this important regulation in the context of infectious diseases. The use of different antibodies against FPN, which are not all carefully characterized by appropriate controls of specificity, could contribute to the differences observed between different studies.

Despite FPN downregulation during *Salmonella* infection in our models, we observed a decrease in iron within infected spleen and liver. Such a decrease in tissues iron was strongly observed in *Hamp*^−/−^ liver but was not seen in AcB61 tissues, likely masked by the exacerbated EP activity and heme iron uptake by macrophages in these tissues. During infection, macrophage iron could be consumed, at least in part, by the bacteria itself, dependent on this metal for its growth and dissemination. Alternatively, FPN-independent export of iron may occur in infected macrophages. A peculiar iron distribution was observed in *Salmonella* infected *Hamp*^−/−^ liver, with some strong accumulation in CL hepatocytes and sinusoidal Kupffer cells. One possible explanation is the engulfment of iron-loaded apoptotic hepatocytes by liver macrophages. Indeed both iron overload and LPS/inflammation have been shown to induce apoptosis in hepatocytes (47, 48). Therefore, in *Hamp*^−/−^ mice, the iron overloaded hepatocytes in the CL zone are likely more sensitive to apoptotic processes during *Salmonella* infection. Since macrophage FPN expression is repressed, an increase of phagocytosis of such apoptotic cells could lead to iron overload in sinusoidal and CL macrophages.

## Conclusion

Our observations suggest that to promote its intracellular growth, *Salmonella* modulates macrophage iron homeostasis to favor its access to intracellular iron with the reduction of iron export *via* the downregulation of FPN. Importantly, such a macrophage cellular host response, which promotes infection, anemia, and hypoferremia, is independent of macrophage iron and HAMP levels. To fight against bacterial infectious diseases and to correct the anemia during chronic infection, effort has to be made to understand whether this HAMP-independent downregulation of FPN expression exists in different macrophages populations and is a general host response observed with other intracellular pathogens infection.

## Ethics Statement

This study was carried out in accordance with the recommendations of the Canadian Council on Animal Care. The protocol was approved by the McGill University Animal Care Committee.

## Author Contributions

AW, SB, ER, and AR designed protocols and performed experiments. AA, RM, and OT performed experiments. SB reviewed data and provided statistical analysis and correction of the manuscript. SV provided *Hamp*^−/−^ mice, reviewed the data, and provided comments and corrections of the manuscript. DM designed protocols, performed experiments, reviewed the data, and provided comments and corrections of the manuscript. FC-H designed protocols, performed experiments, reviewed the data, and wrote the paper.

## Conflict of Interest Statement

The authors declare that the research was conducted in the absence of any commercial or financial relationships that could be construed as a potential conflict of interest. The handling editor declared a shared affiliation, though no other collaboration, with several of the authors (AW, AA, and FC-H), and states that the process nevertheless met the standards of a fair and objective review.
