# Supplementary material for: Iron- and Hepcidin-Independent Downregulation of the Iron Exporter Ferroportin in Macrophages during Salmonella Infection
Source: Front Immunol. 2017 May 1;8:498. doi: 10.3389/fimmu.2017.00498 (PMC5410627; doi:10.3389/fimmu.2017.00498)
Supplement: Supplementary file 1 [file Presentation_1.PDF]

S4 Extended Materials and Method

Animals and *Salmonella* infection *in vivo*

All animal experiments were done under conditions specified by the Canadian Council on Animal Care and protocols approved by the McGill University Animal Care Committee. The generation of AcB61 has been reported previously [1]. A/J mice were purchased from the Jackson Laboratory. $Hamp^{tm1Svl}$ knockout mice (*Hamp*$^{-/-}$ [2]) were transferred onto a 129S6 background (129S6.B6*129S2-*Hamp*^{tm1Svl}), a strain highly resistant to *Salmonella* Typhimurium infection [3]. *In vivo Salmonella* infections were performed as previously described using either *Salmonella enterica* serovar Typhimurium strain Keller [6]. In brief, *Salmonella* were grown in trypticase soy broth, and each mouse was infected intravenously with ~1000 CFUs (A/J and AcB61) and ~5000 CFUs (WT and *Hamp* diluted in 200 µl of 0.9% saline. The infectious dose was verified by plating of serial dilutions on trypticase soy agar. Bacterial loads in the spleen and liver were determined by plating serial dilutions of organ homogenates on trypticase soy agar. All mice were aged between 8-12 weeks. The spleen index was calculated as the root square of spleen weight (x100) divided by body weight [7].

Bone Marrow Derived Macrophage cultures *Salmonella* infection *in vitro*

Murine bone marrow derived macrophages (BMDM) were cultured as described previously [8, 9]. Briefly, bone marrow cells were isolated from femurs of SWISS CD1 washed in HBSS and seeded at a concentration of $3x10^5$ cells/mL in complete medium (RPMI 1640 Glutamax medium supplemented with 10 % (v/v) fetal bovine serum (FBS), 10 % (v/v) L929-conditioned culture medium, 1 % (v/v) penicillin and streptomycin) and incubated at 37 ºC with 5 % CO2. At day 4, adherent cells were washed with warm HBSS and remained

in culture with daily medium renewal until infection at day 8 of culture of BMDM. For *in vitro* infection of macrophages, *Salmonella* Typhimurium SL1344 [10] was grown with agitation at 37°C in Tryptic soy broth (TSB) to either a log logarithmic phase (exponential phase) or a stationary phase (overnight culture). Prior to infection, BMDM were cultured 16hr in RPMI 1640 with 2% (v/v) FBS serum and then infected with *Salmonella* for 1h with a MOI (Multiplicity of infection) of between 5 and 10. Bacteria were then removed and the remaining extracellular bacteria were killed by incubation with 100 µg/ml gentamicin in fresh medium for 1hr. Cells were then washed and cultured in fresh medium containing 10 µg/ml of gentamicin until the time points of RNA extraction.

Blood parameters analysis

Mice were humanely euthanized by CO_2 asphyxiation at various time points; blood was collected by cardiac puncture and immediately transferred to pediatric 200µl EDTA tubes. Hematology profiles were performed at the McGill Comparative Medicine and Animal Resources Centre (Montréal, QC, Canada). SerumPlasma iron, ferritin, transferrin and bilirubin levels were measured with an Olympus AU400 automat at the Laboratory of Biochemistry at the Institut Fédératif de Recherche 02, CHU Bichat-Claude Bernard (Paris, France).

Tissues iron studies

Liver and spleen iron contents were determined by acid digestion of tissue samples, as described by Torrance and Bothwell [11], followed by determination of the iron content in the tissue lysate using an Olympus AU400 automat. For tissue iron staining, spleen and liver were obtained from A/J and AcB61 mice, fixed overnight in 10% formalin (neutral-buffered), dehydrated in ethanol/xylene and embedded in paraffin wax. Histological sections were cut on a microtome and fixed to glass slides. Sections were deparaffinized in xylene,

rehydrated in a series of ethanol baths, and then processed for intracellular iron staining using Perl's solution. After iron staining, sections were then stained with hematoxylin/eosin, dehydrated in ethanol/xylene and mounted with Permount. Stained sections were examined under a light microscope and photographed or digitalized using a slide scanner (Panoramic 250 from 3DHISTEC).

Antibodies

Polyclonal rabbit antisera against FPN were produced and affinity purified as previously described [12, 13]. The specificity of this serum was established by immunoblotting, immunofluorescence and histochemistry [12, 14, 15]. The rabbit polyclonal anti-Human Heme oxygenase 1 (HMOX1) was from Stressgen Biotechnologies Corp (Victoria, BC Canada). The mouse monoclonal anti-vinculin was purchased from Sigma–Aldrich. The secondary peroxidase antibody anti-mouse, anti-rabbit and anti-rat were purchased from DAKO (town and country). The goat anti-rabbit Ig–alexa 488 (GAR-alexa488) and the goat anti-Rat Ig-alexa 568 (GAR-alexa568) secondary antibodies were from MolecularProbes.

Immunohistofluorescence studies

Tissues sections from spleen and liver were deparaffinized in xylene and rehydrated in a series of ethanol baths before incubation in a blocking solution (BSA 1% and 10% heat inactivated goat serum in PBS) for 30 min at room temperature. Incubation with primary antibodies was then performed in a humid chamber at room temperature for 1hr using the following dilution in blocking solution: rabbit anti-FPN: 1/50 to 1/100; rabbit anti-HMOX1: 1/500; Mouse anti- F4/80 1/500. After three washes with PBS/0.5% BSA, cells were incubated for 1hr at RT with GAR-alexa488 (1/200) and GAR-Alexa568 (1/200) in blocking solution. Slide coverslips were then washed 3X in PBS/0.5% BSA, 1X in PBS, mounted with

antifading mounting reagent (Prolong Antifade kit P-7481 MolecularProbes) and processed for microscopy. Cells were visualized using either an epifluorescence microscope LEICA DM-IRM with a 10X, 20X, 40X and a 100X oil immersion objective or a Zeiss confocal fluorescent microscope with a 60X oil immersion objective. Images were acquired using either ARCHIMED-PRO (Microvision Instruments) or Zeiss LSM Image Browser softwares. Red blood cells (RBC) were red/orange when visualized using the autofluorescence of hemoglobin (emission at 520nm).

Western blot analysis

Crude membrane fractions from mouse tissues were prepared as previously described [12]. Proteins (40µg for spleen and 80µg for liver) were solubilized in 1X Laemmli buffer and incubated for 30 min at room temperature (RT), prior to SDS PAGE and transfered on to PVDF. Ponceau red staining confirmed similar gel loading and similar transfer of proteins to the membranes. Immunoblots were pre-incubated with blocking solution [7% skim milk in TBST (0.15% Tween20 in Tris buffered saline)] for 16 hrs at 4°C prior to incubation with primary antibodies for 2 hrs at RT. Antibodies were diluted in blocking solution as follows: anti-FPN : 1/200 (liver) or 1/500 (spleen), anti-HMOX1 : 1/4000, anti-Lamp1 1/500 and anti-TfR1 : 1/200. After incubation with antibodies, membranes were washed 6 times (5 min each) in TBST and then incubated with peroxidase-labeled secondary antibodies (1/50000) for 1 hr at RT. Signals were visualized by enhanced chemiluminescence (ECL, Millipore). PVDF membranes were stripped (100 mM/L β2-mercaptoethanol, 2% SDS, 62.5 mmol/L Tris-HCL pH 6.8; 50°C, 30 min) and then re-probed with a different primary antibody.

RNA studies from mouse tissues

Total RNA was extracted from the liver with Trizol Reagent (Life Technologies Inc.) according to the manufacturer instructions. cDNAs were synthesized all at once using M-

MLV reverse transcriptase (Invitrogen). Quantitative PCR was performed on Chromo4 Real-Time PCR Detection System (Bio-Rad Laboratories) using Brilliant SYBR Green QPCR Master Mix (Stratagene). All samples were amplified in duplicates during the same PCR run on 96 wells plates. The relative expression of the genes was normalized to the amount of *Tbp* (TATA box binding protein) or *Hprt* (hypoxanthine guanine phosphoribosyl transferase) and relative to the average expression of the reference group (uninfected A/J for AcB61 mice or uninfected 129S6 or C57BL/6 wildtype mice for Hamp$^{-/-}$) using the comparative ΔΔCt method. Gene expression fold changes were calculated using the formula $2^{-\Delta\Delta Ct}$, in which $\Delta\Delta Ct^{A\text{-}B}$= $(Ct^{gene}$- $Ct^{Hprt})$ B -$(Ct^{gene}$- $Ct^{Hprt})$ A and A= WT and B=*Hamp*$^{-/-}$. Data are presented as fold changes ($2^{-\Delta\Delta Ct}$) in infected mice relative to the mean value of A/J or wildtype (control) at each time point.

RNA studies from macrophage cultures

TRIzol reagent (Invitrogen) was added directly to cell culture plates after discarding culture medium. Cell lysates were homogenized and stored at -80 ºC until RNA extraction. Total RNA was extracted according to manufacturer's instructions, followed by quantitative and qualitative analysis using Nanophotometer P360 (Implen). After synthesis of cDNA with MMLV Reverse Transcriptase (Invitrogen), quantitative PCR (qPCR) was performed on a LightCycler 480 instrument (Roche Diagnostics). Melting curve analysis of amplified products was performed for confirmation of primers' specificity. In all experiments, every sample was run in duplicate (technical replicate). For kinetic studies, all time points were tested in biological triplicates (biological replicate). The gene *Hprt* was used as reference gene and relative gene expression were expressed as –ΔCT (CT gene of interest-CT *Hprt*).

Online Supplement References

1. Fortin A, Diez E, Rochefort D, Laroche L, Malo D, Rouleau GA, et al. Recombinant congenic strains derived from A/J and C57BL/6J: a tool for genetic dissection of complex traits. Genomics. 2001;74(1):21-35.
2. Lesbordes-Brion JC, Viatte L, Bennoun M, Lou DQ, Ramey G, Houbron C, et al. Targeted disruption of the hepcidin 1 gene results in severe hemochromatosis. Blood. 2006;108(4):1402-5.
3. Roy MF, Malo D. Genetic regulation of host responses to Salmonella infection in mice. Genes Immun. 2002;3(7):381-93.
4. Caron J, Lariviere L, Nacache M, Tam M, Stevenson MM, McKerly C, et al. Influence of Slc11a1 on the outcome of Salmonella enterica serovar Enteritidis infection in mice is associated with Th polarization. Infect Immun. 2006;74(5):2787-802.
5. Caron J, Loredo-Osti JC, Laroche L, Skamene E, Morgan K, Malo D. Identification of genetic loci controlling bacterial clearance in experimental Salmonella enteritidis infection: an unexpected role of Nramp1 (Slc11a1) in the persistence of infection in mice. Genes Immun. 2002;3(4):196-204.
6. Roy MF, Riendeau N, Loredo-Osti JC, Malo D. Complexity in the host response to Salmonella Typhimurium infection in AcB and BcA recombinant congenic strains. Genes Immun. 2006;7(8):655-66.
7. Forget A, Skamene E, Gros P, Miailhe AC, Turcotte R. Differences in response among inbred mouse strains to infection with small doses of Mycobacterium bovis BCG. Infect Immun. 1981;32(1):42-7.
8. Delaby C, Pilard N, Hetet G, Driss F, Grandchamp B, Beaumont C, et al. A physiological model to study iron recycling in macrophages. Exp Cell Res. 2005;310(1):43-53.
9. Delaby C, Pilard N, Hetet G, Driss F, Grandchamp B, Beaumont C, et al. A physiological model to study iron recycling in macrophages. Exp Cell Res. 2005;310(1):43-53.
10. Hoiseth SK, Stocker BA. Aromatic-dependent Salmonella typhimurium are non-virulent and effective as live vaccines. Nature. 1981;291(5812):238-9.
11. Torrance JD, Bothwell TH. A simple technique for measuring storage iron concentrations in formalinised liver samples. S Afr J Med Sci. 1968;33(1):9-11.
12. Canonne-Hergaux F, Donovan A, Delaby C, Wang HJ, Gros P. Comparative studies of duodenal and macrophage ferroportin proteins. Am J Physiol Gastrointest Liver Physiol. 2006;290(1):G156-63.
13. Canonne-Hergaux F, Gruenheid S, Ponka P, Gros P. Cellular and subcellular localization of the Nramp2 iron transporter in the intestinal brush border and regulation by dietary iron. Blood. 1999;93(12):4406-17.
14. Auriac A, Willemetz A, Canonne-Hergaux F. Lipid raft-dependent endocytosis: a new route for hepcidin-mediated regulation of ferroportin in macrophages. Haematologica. 2010;95(8):1269-77.
15. Delaby C, Pilard N, Goncalves AS, Beaumont C, Canonne-Hergaux F. Presence of the iron exporter ferroportin at the plasma membrane of macrophages is enhanced by iron loading and down-regulated by hepcidin. Blood. 2005;106(12):3979-84.
