# Supplementary figures and images for: Iron- and Hepcidin-Independent Downregulation of the Iron Exporter Ferroportin in Macrophages during Salmonella Infection
Source: Front Immunol. 2017 May 1;8:498. doi: 10.3389/fimmu.2017.00498 (PMC5410627; doi:10.3389/fimmu.2017.00498)

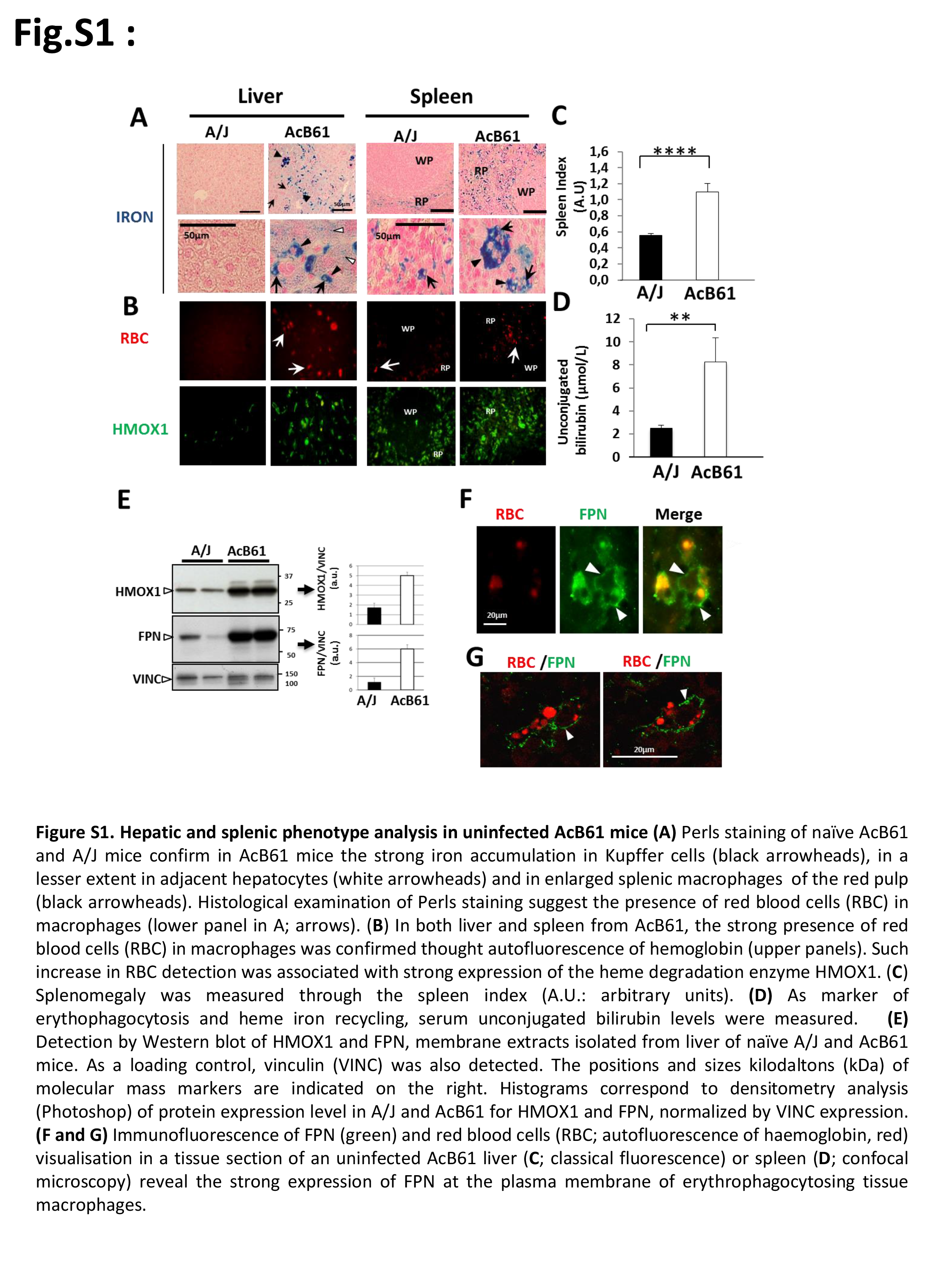

Supplement: Supplementary file 2 [file Image_1.TIF]

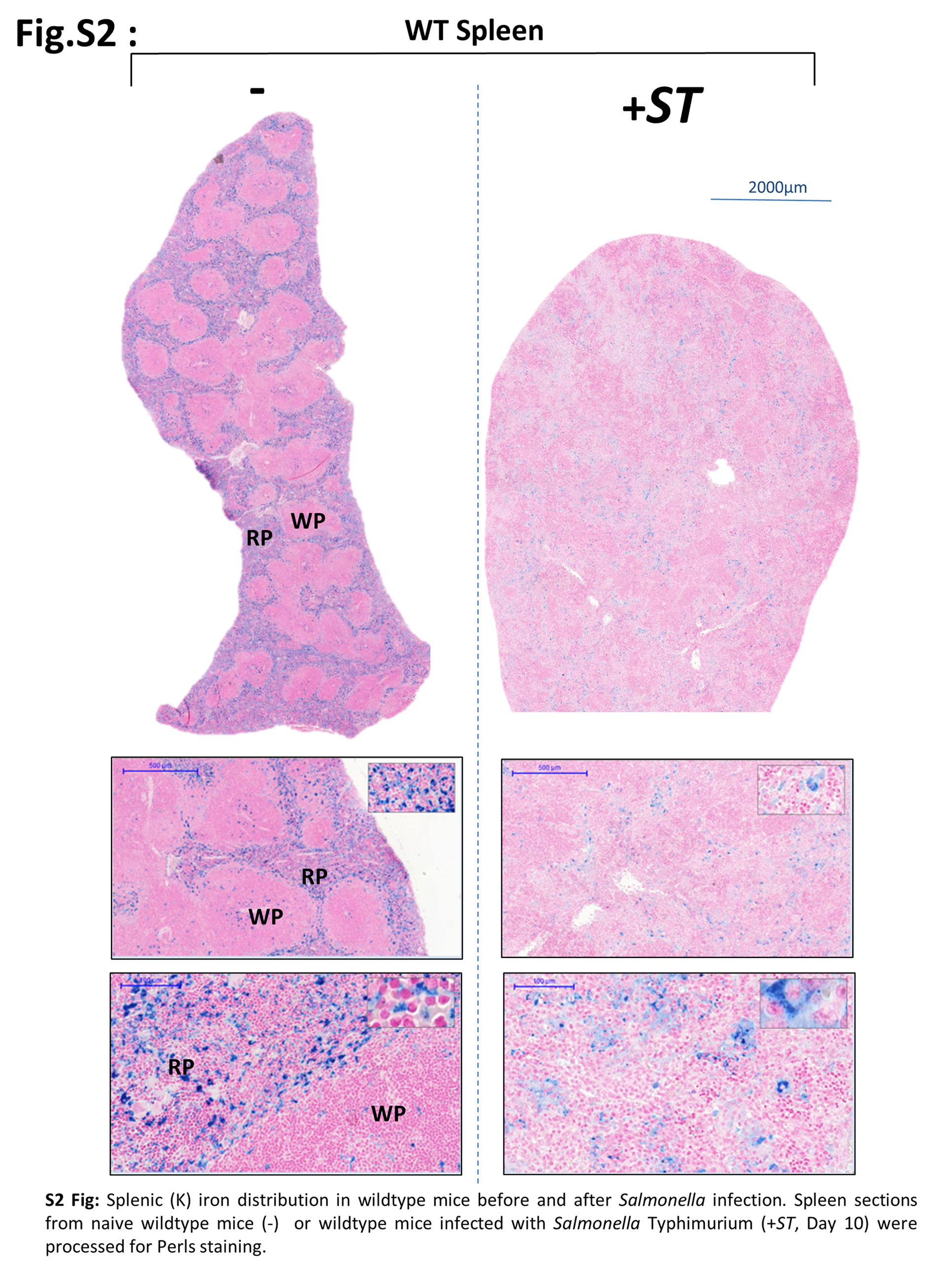

Supplement: Supplementary file 3 [file Image_2.TIF]

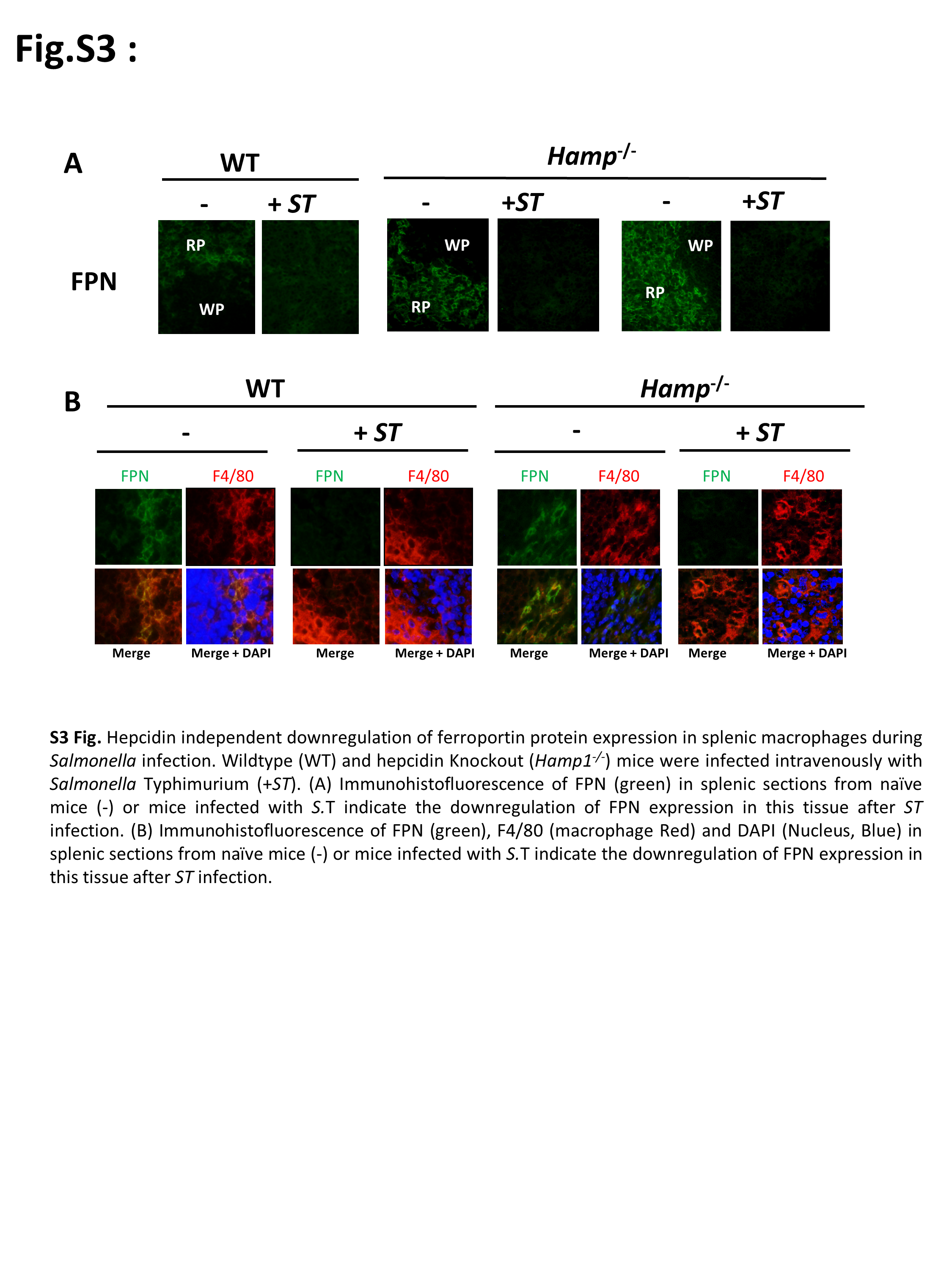

Supplement: Supplementary file 4 [file Image_3.TIF]

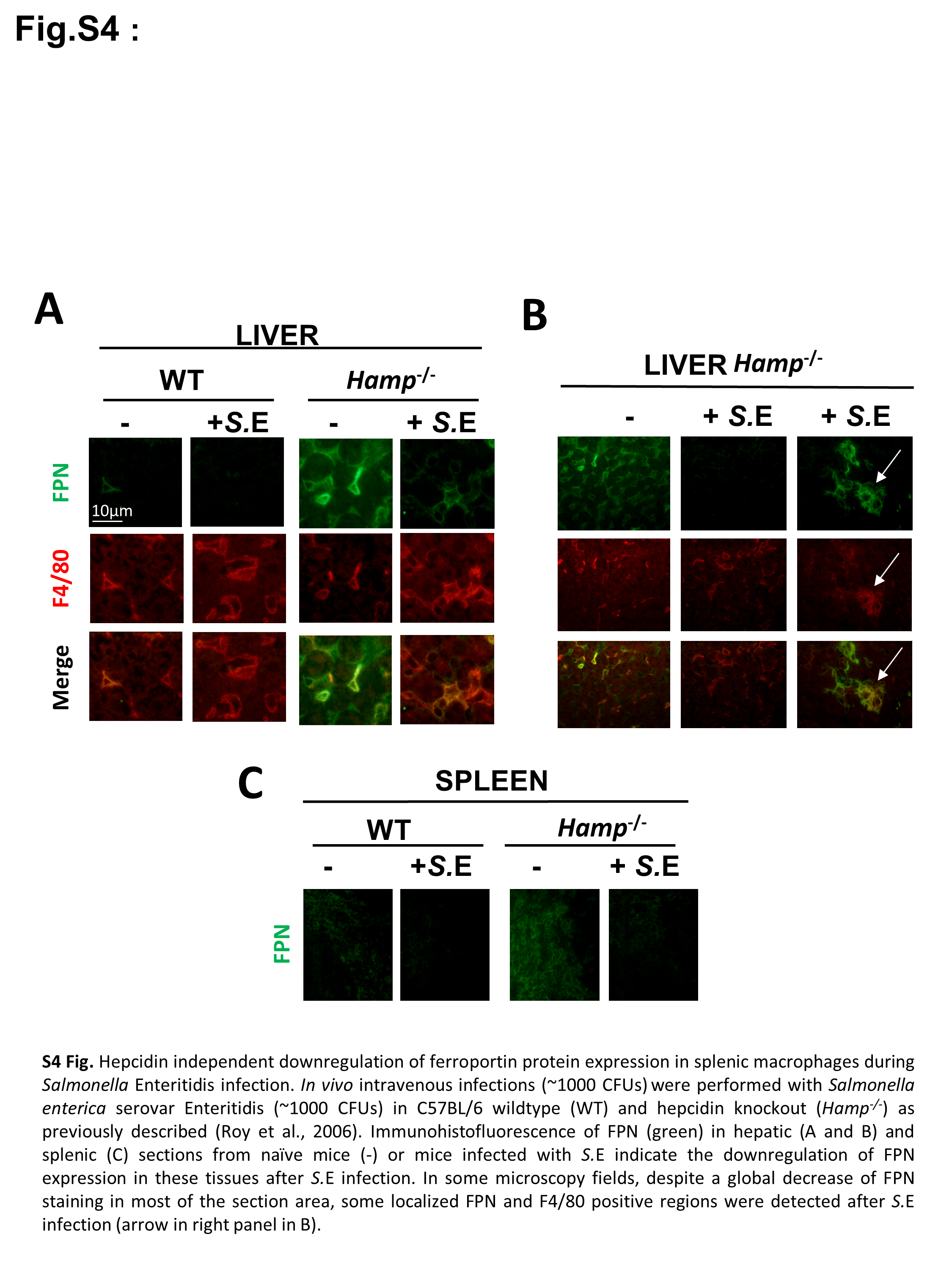

Supplement: Supplementary file 5 [file Image_4.TIF]

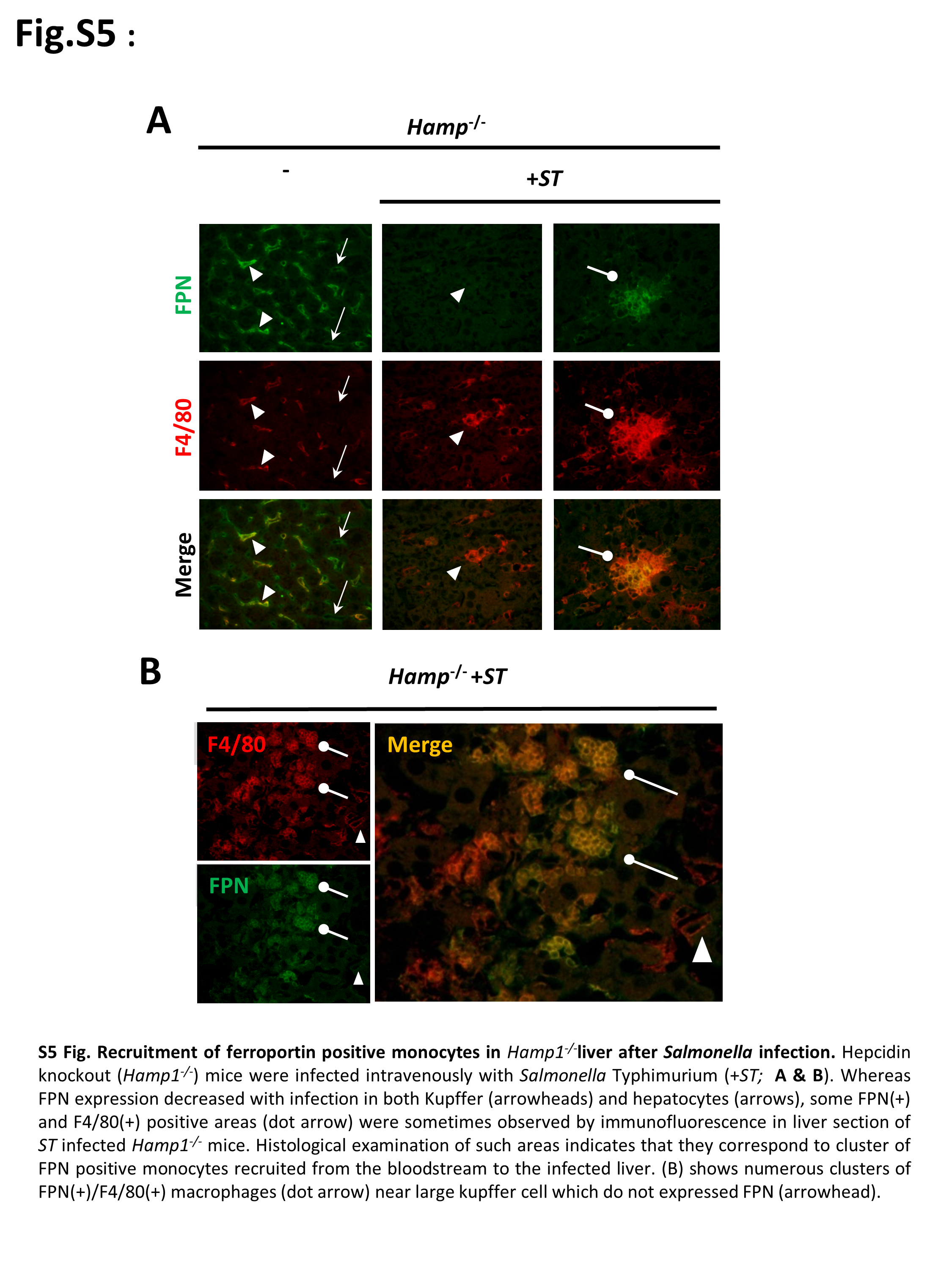

Supplement: Supplementary file 6 [file Image_5.TIF]
